# Changes in antimicrobial resistance of staphylococci isolated from bloodstream infection and the impact of COVID-19

**DOI:** 10.1186/s12879-026-13949-5

**Published:** 2026-07-08

**Authors:** Yan Wu, Mengjun Zhou

**Affiliations:** 1 Department of Clinical Laboratory, Mianyang Central Hospital, School of Medicine, University of Electronic Science and Technology of China, Mianyang, 621000 China; 2 Medical Insurance Department, Mianyang Central Hospital, School of Medicine, University of Electronic Science and Technology of China, Mianyang, 621000 China

**Keywords:** Bloodstream infection, *Staphylococcus aureus*, Coagulase-negative staphylococci, Antimicrobial resistance, COVID-19, Trend analysis

## Abstract

**Objective:**

To analyze the changes in antimicrobial resistance of *Staphylococcus aureus* (*S. aureus*) and coagulase-negative staphylococci (CoNS) in bloodstream infections (BSIs) between 2018 and 2024, and to assess temporal associations with the COVID-19 pandemic.

**Methods:**

A total of 575 *S. aureus* and 1,014 CoNS isolates from blood cultures were retrospectively reviewed. To define true CoNS BSI, we applied clinical criteria (≥ 2 positive bottles with same species, or 1 positive bottle with clinical signs + central line + targeted therapy). Demographic characteristics and resistance rates to 15 antimicrobial agents were compared using chi-square (χ^2^) and Mann-Kendall tests, with 95% confidence intervals (CIs) and Bonferroni correction for multiple comparisons.

**Results:**

CoNS infections were more common in minors (0–17 years: 31.3%) and the elderly (≥ 80 years: 13.7%), while *S. aureus* predominated in adults 18–65 years (51.8%). CoNS exhibited higher resistance rates than *S. aureus* to oxacillin (75.2% vs. 30.5%, *p* < 0.001), ciprofloxacin (42.8% vs. 17.5%, *p* < 0.001) and erythromycin (82.2% vs. 56.1%, *p* < 0.001). Methicillin-resistant CoNS (MRCNS) detection rates exceeding 70% throughout. Penicillin G resistance in *S. aureus* increased from 87.1% (95% CI 78.0–93.0) in 2018 to 100% (95% CI 92.5–100) in 2024 (nominal *P* < 0.05, but not significant after correction). Following the pandemic onset, resistance rates to penicillin G, clindamycin, and ciprofloxacin further increased in both groups. After Bonferroni correction, the pandemic-period resistance rate increases of penicillin G, oxacillin, clindamycin, and erythromycin for *S. aureus* remained significant, while those for CoNS did not. No resistance to linezolid, vancomycin, or tigecycline was detected.

**Conclusion:**

Staphylococci resistance in blood isolates has intensified, with temporal associations observed during the Coronavirus Disease 2019 (COVID-19) pandemic. Clinicians should reconsider empirical oxacillin use given persistently high methicillin resistance. Ongoing surveillance and antimicrobial stewardship are essential. Causal attribution is not possible due to uncontrolled confounders.

## Introduction

Bloodstream infections (BSIs) are common and severe infectious diseases in clinical practice [1, 2]. Staphylococci are frequent pathogens in both healthcare-associated and community-acquired infections. Among them, *Staphylococcus aureus* (*S. aureus*) and coagulase-negative staphylococci (CoNS) are the two most commonly isolated species in clinical settings and represent major causative agents of BSIs [3]. With the widespread use of antimicrobial agents, drug resistance in staphylococci has become an increasingly serious problem. The emergence of methicillin-resistant strains, specifically methicillin-resistant *S. aureus* (MRSA) and methicillin-resistant CoNS (MRCNS), has significantly increased treatment difficulties and poses a major challenge to clinical management [4, 5]. This study aims to analyze the resistance data of *S. aureus* and CoNS isolates from BSIs to 15 commonly used antimicrobial agents between 2018 and 2024. The objective is to elucidate the temporal trends in antimicrobial resistance of these two staphylococcal groups and to assess temporal associations with the COVID-19 pandemic.

## Subjects and methods

### Study subjects and definitions

Clinical isolates were collected from a tertiary hospital (Northwest Sichuan Regional Medical Center) between January 2018 and December 2024. All positive blood cultures were reviewed. Inclusion criteria: (i) patient age ≥ 1 day, (ii) first isolate per patient per species per year. Exclusion criteria: duplicate isolates from same patient within 30 days.

For CoNS, true bloodstream infection was defined using the following clinical and microbiological criteria: (i) ≥ 2 blood culture bottles positive for the same CoNS species within 48 h, OR (ii) One positive bottle plus at least two of the following: body temperature > 38℃ or < 36℃, white blood cell count > 12,000/µL or < 4,000/µL, C-reactive protein > 50 mg/L, presence of central venous catheter, and initiation of targeted antimicrobial therapy by the attending physician. Isolates not meeting these criteria were considered contaminants and excluded. For *S. aureus*, a single positive bottle with clinical signs was considered significant, given its lower contamination rate. After exclusions, 575 *S. aureus* and 1,014 CoNS isolates were included.

**Fig. 1:**
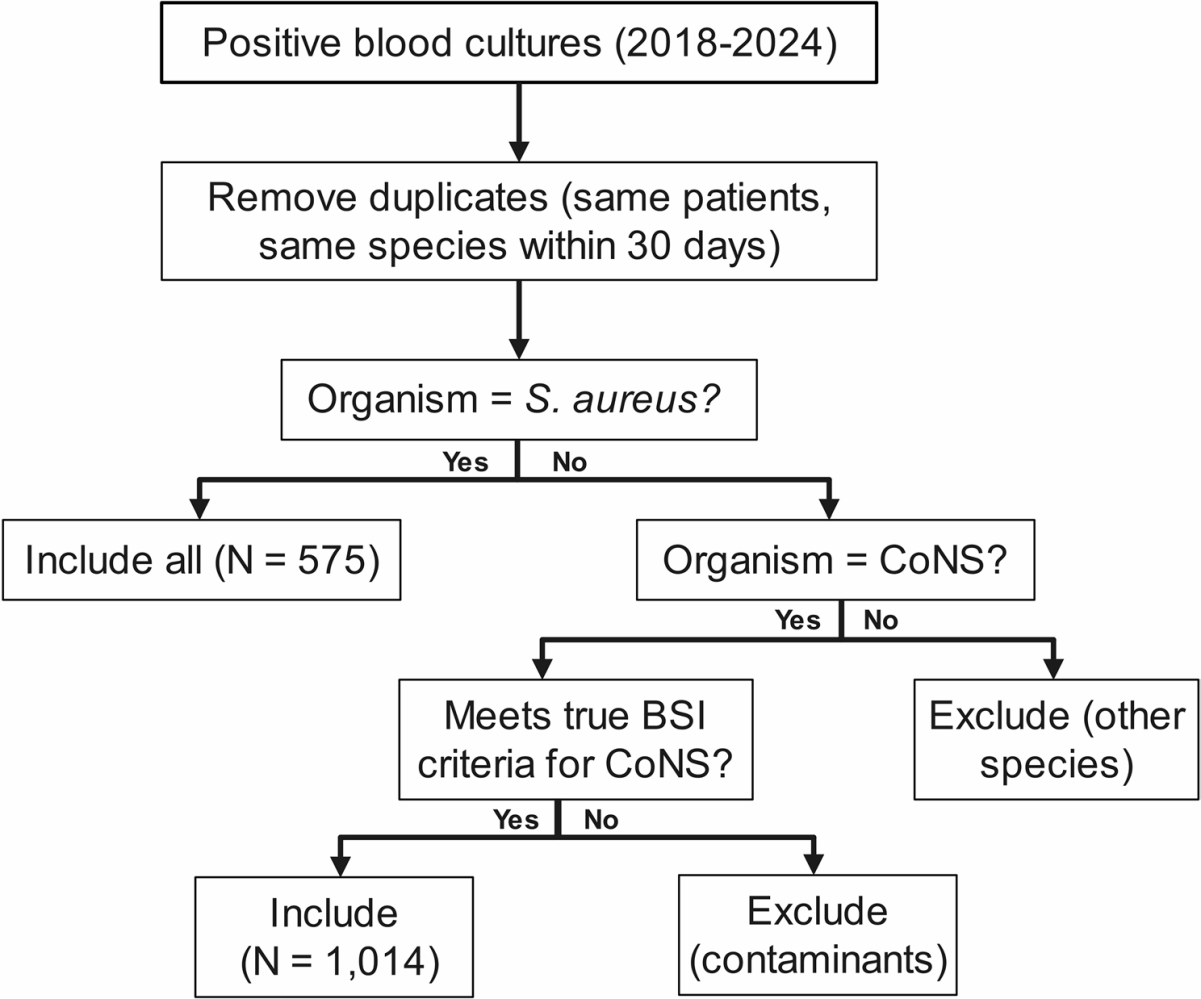
Flow chart of study. From 2018 to 2024, a total of 1,589 blood culture positive patients were analyzed for antibacterial resistance, including 575 cases of *S.aureus* infection and 1,014 cases of CoNS infection. True BSI criteria for CoNS: (**a**) ≥ 2 positive bottles same species, OR (**b**) 1 bottle + clinical signs + central line + targeted therapy

The study was approved by the Ethics Committee Office of Mianyang Central Hospital (No. 2S202403146-01) and adhered to the Helsinki Declaration. Informed consent was waived for anonymized routine data. The study flow is presented in Fig. 1.

### Bacterial culture and identification

Blood cultures were performed using the Bact/Alert 3D system (BioMérieux, Marcy l Etoile, France) and the BacT/ALERT^®^ TIRTUO™ automated microbial culture system (BioMérieux). When a positive signal was detected, the broth was aseptically aspirated and inoculated onto blood agar and chocolate agar plates, then incubated at 35 ℃ in a CO₂ incubator for 18 to 24 h. Bacterial identification methods: VITEK 2 Compact system (BioMérieux) was used throughout. In 2019, the laboratory additionally introduced VITEK^®^ MS matrix-assisted laser desorption/ionization time-of-flight (MALDI-TOF) mass spectrometry (BioMérieux) for identification.

### Antimicrobial susceptibility testing

Susceptibility testing was performed using VITEK 2 Compact cards (BioMérieux). Results were interpreted according to Clinical and Laboratory Standards Institute (CLSI) guidelines of each respective year (versions: 2018–2024). Nitrofurantoin was excluded from analysis because it is not indicated for bloodstream infections.

### Statistical analysis

Annual resistance rates were analyzed using WHONET 5.6. Comparative analyses were performed with SPSS 26.0. All resistance proportions are reported with 95% confidence intervals (CIs) calculated using the Wilson method. Comparisons between *S. aureus* and CoNS used chi-square (*χ2*) tests. For comparisons across three periods (pre-pandemic: 2018–2019, pandemic: 2020–2022, post-pandemic: 2023–2024), the Cochran-Armitage test for trend was used. For annual trends, the Mann-Kendall test was applied, supplemented by linear regression on logit-transformed proportions. To control for multiple comparisons across 15 antimicrobial agents, Bonferroni correction was applied (adjusted α = 0.05/15 = 0.003 for Tables 2, 3 and 4; for Tables 5 and 6, the same correction was applied per table). A *P*-value ≤ 0.05 before correction was considered nominally significant, but only corrected *P*-values below 0.003 are considered statistically significant in the interpretation.

**Table 1:** Gender and age distribution of the included patients

Characteristic	*S. Aureus* (*N* = 575)	CoNS (*N* = 1,014)	χ²	*P*
**Gender**,** N (%)**			1.430	0.232
Male	355 (61.7%, 95% CI 57.7–65.6)	595 (58.7%, 95% CI 55.6–61.7)		
Female	220 (38.3%, 95% CI 34.4–42.3)	419 (41.3%, 95% CI 38.3–44.4)		
**Age distribution**,** N (%)**			120.679	< 0.001
0–17 years (pediatric)	80 (13.9%, 95% CI 11.2–17.0)	317 (31.3%, 95% CI 28.5–34.2)		
18–65 years (adult)	298 (51.8%, 95% CI 47.7–55.9)	284 (28.0%, 95% CI 25.3–30.9)		
66–79 years (elderly)	162 (28.2%, 95% CI 24.6–32.0)	274 (27.0%, 95% CI 24.3–29.9)		
≥ 80 years (very elderly)	35 (6.1%, 95% CI 4.3–8.4)	139 (13.7%, 95% CI 11.7–16.0)		

Data are presented as number (percentage, 95% confidence interval). Percentages may not sum to 100% due to rounding. Age groups are interpreted clinically as pediatric (0–17 years), adult (18–65 years), elderly (66–79 years), and very elderly (≥ 80 years). ***χ²*** test was used for comparison between *S. aureus* and CoNS groups

**Table 2:** Resistance percentages of *S. aureus* and CoNS isolates to 15 antimicrobial agents (2018–2024)

Antimicrobial agent	*S. aureus* (*N* = 575)	CoNS (*N* = 1,014)	χ²	*P*
Penicillin G	92.5% (90.0–94.5)	96.4% (95.2–97.4)	1.451	0.228
Oxacillin	30.5% (26.8–34.4)	75.2% (72.5–77.8)	40.092	< 0.001*
Gentamicin	3.7% (2.3–5.6)	10.0% (8.2–12.0)	3.110	0.078
Rifampicin	0.4% (0.1–1.4)	6.9% (5.4–8.7)	6.007	0.014
Ciprofloxacin	17.5% (14.5–20.9)	42.8% (39.8–45.9)	15.197	< 0.001*
Levofloxacin	17.4% (14.4–20.8)	46.5% (43.5–49.6)	19.474	< 0.001*
Moxifloxacin	15.8% (13.0–19.1)	26.8% (24.1–29.7)	3.609	0.057
Cotrimoxazole	10.8% (8.5–13.6)	57.0% (53.9–60.0)	47.627	< 0.001*
Clindamycin	29.4% (25.8–33.3)	31.9% (29.1–34.8)	0.147	0.701
Erythromycin	56.1% (52.0–60.2)	82.2% (79.8–84.5)	15.966	< 0.001*
Linezolid	0% (0–0.6)†	0% (0–0.4)†	–	–
Vancomycin	0% (0–0.6)†	0% (0–0.4)†	–	–
Quinupristin/dalfopristin	0% (0–0.6)†	0.5% (0.2–1.2)	0.522	0.470
Tetracycline	19.1% (16.0–22.6)	19.8% (17.4–22.5)	0.016	0.901
Tigecycline	0% (0–0.6)†	0% (0–0.4)†	–	–

Data are presented as % (95% CI). After Bonferroni correction for 15 comparisons (α = 0.003), oxacillin, ciprofloxacin, levofloxacin, cotrimoxazole, and erythromycin remained significant. † For zero events, the upper bound of 95% CI estimated by rule of three. “–” indicates *χ*^*2*^ not calculated. Nitrofurantoin excluded

**Table 3:** Annual resistance rates of *S. aureus* to 15 antimicrobial agents, 2018–2024

Antimicrobial agent	2018 (*N* = 79)	2019 (*N* = 58)	2020 (*N* = 107)	2021 (*N* = 117)	2022 (*N* = 122)	2023 (*N* = 45)	2024 (*N* = 47)	Predicted 2018	Predicted 2024	Slope (%/year)	Z	P*
Penicillin G	87.1 (78.0–93.0)	86.4 (75.3–93.1)	94.2 (88.1–97.3)	93.1 (87.1–96.5)	91.6 (85.4–95.4)	100 (92.5–100)†	100 (92.5–100)†	86.5 (82.1–90.9)	99.4 (94.8–104.0)	2.15 (0.62–3.68)	1.652	0.049‡
Oxacillin	8.6 (3.8–17.1)	20.3 (11.8–32.5)	44.2 (35.0–53.8)	41.6 (32.9–50.8)	27.7 (20.3–36.4)	36.4 (23.6–51.4)	39.1 (26.4–53.5)	13.2 (7.3–19.1)	36.7 (27.9–45.5)	3.92 (1.15–6.69)	0.901	0.184
Gentamicin	5.4 (1.8–13.2)	10.2 (4.2–21.5)	5.8 (2.5–11.7)	0 (0–3.1)†	5.9 (2.6–11.7)	0 (0–7.9)†	6.5 (1.9–17.8)	6.8 (2.9–10.7)	5.3 (0.6–10.0)	-0.25 (-1.21–0.71)	0	0.500
Rifampicin	0% (0–4.6)†	5.1 (1.4–14.1)	3.8 (1.3–9.2)	0 (0–3.1)†	1.7 (0.3–6.0)	0 (0–7.9)†	0 (0–7.6)†	2.9 (0.1–5.7)	0.0 (-3.1–3.1)	-0.48 (-1.37–0.41)	-0.901	0.184
Ciprofloxacin	17.2 (10.3–27.1)	27.1 (17.3–39.6)	7.7 (3.7–14.4)	24.8 (17.7–33.5)	23.5 (16.7–32.0)	11.4 (4.5–24.3)	17.4 (8.8–30.9)	20.2 (12.8–27.6)	18.3 (9.5–27.1)	-0.32 (-2.10–1.46)	-0.300	0.382
Levofloxacin	17.2 (10.3–27.1)	27.1 (17.3–39.6)	7.7 (3.7–14.4)	24.8 (17.7–33.5)	23.5 (16.7–32.0)	9.1 (3.1–21.6)	17.4 (8.8–30.9)	20.2 (12.8–27.6)	18.3 (9.5–27.1)	-0.32 (-2.10–1.46)	-0.300	0.382
Moxifloxacin	17.2 (10.3–27.1)	25.4 (15.8–38.0)	7.7 (3.7–14.4)	24.8 (17.7–33.5)	20.2 (13.8–28.5)	4.5 (0.8–15.5)	10.9 (4.1–23.6)	20.0 (12.5–27.5)	12.6 (3.7–21.5)	-1.23 (-2.98–0.52)	-0.901	0.184
Cotrimoxazole	11.0 (5.5–20.4)	13.8 (6.9–25.1)	14.4 (8.9–22.2)	16.8 (10.8–24.9)	13.4 (8.5–20.3)	4.5 (0.8–15.5)	8.7 (3.0–20.0)	12.9 (7.3–18.5)	9.7 (2.8–16.6)	-0.53 (-1.61–0.55)	-0.601	0.274
Clindamycin	15.1 (8.6–25.0)	23.7 (14.2–36.7)	42.3 (33.2–51.9)	51.5 (42.3–60.6)	16.0 (10.2–23.9)	22.7 (12.6–37.5)	34.8 (22.4–49.5)	24.3 (15.5–33.1)	31.3 (18.5–44.1)	1.17 (-1.86–4.20)	0.601	0.274
Erythromycin	41.9 (31.4–53.2)	50.8 (37.9–63.7)	67.3 (57.8–75.6)	71.3 (62.4–78.9)	52.9 (44.0–61.6)	59.1 (44.2–72.6)	52.2 (37.9–66.1)	49.8 (40.0–59.6)	56.5 (41.9–71.1)	1.12 (-2.65–4.89)	0.601	0.274
Linezolid	0 (0–4.6)†	0 (0–6.2)†	0 (0–3.4)†	0 (0–3.1)†	0 (0–3.0)†	0 (0–7.9)†	0 (0–7.6)†	0 (0–0)	0 (0–0)	0 (0–0)	0	0.500
Vancomycin	0 (0–4.6)†	0 (0–6.2)†	0 (0–3.4)†	0 (0–3.1)†	0 (0–3.0)†	0 (0–7.9)†	0 (0–7.6)†	0 (0–0)	0 (0–0)	0 (0–0)	0	0.500
Quinupristin/dalfopristin	0 (0–4.6)†	0 (0–6.2)†	0 (0–3.4)†	0 (0–3.1)†	0 (0–3.0)†	0 (0–7.9)†	0 (0–7.6)†	0 (0–0)	0 (0–0)	0 (0–0)	0	0.500
Tetracycline	19.4 (11.9–29.8)	30.5 (19.9–43.5)	14.4 (8.9–22.2)	24.8 (17.7–33.5)	25.2 (18.2–33.7)	15.9 (7.4–30.2)	23.9 (13.6–38.5)	23.5 (16.9–30.1)	21.6 (13.6–29.6)	-0.32 (-1.96–1.32)	0	0.500
Tigecycline	0 (0–4.6)†	0 (0–6.2)†	0 (0–3.4)†	0 (0–3.1)†	0 (0–3.0)†	0 (0–7.9)†	0% (0–7.6)†	0 (0–0)	0 (0–0)	0 (0–0)	0	0.500

Data are presented as % (95% CI). Linear regression was performed with year as continuous predictor and resistance percentage as outcome. Slope represents average annual change in percentage points. Predicted values and 95% CIs are derived from the regression model. Mann-Kendall Z and nominal *P*-values are shown for trend robustness. † For 0% or 100% events, the 95% CI upper or lower bound is estimated by the rule of three. ‡ After Bonferroni correction for 15 comparisons (α = 0.05/15 = 0.003), no trend reached statistical significance. The nominal *P*-value for penicillin G (0.049) is not significant after correction. All other nominal *P*-values are > 0.05. Nitrofurantoin was excluded because it is not indicated for bloodstream infections

**Table 4:** Annual resistance rates of CoNS to 15 antimicrobial agents, 2018–2024

Antimicrobial agent	2018 (*N* = 101)	2019 (*N* = 71)	2020 (*N* = 142)	2021 (*N* = 174)	2022 (*N* = 171)	2023 (*N* = 202)	2024 (*N* = 153)	Predicted 2018	Predicted 2024 ]	Slope (%/year) ]	Z	P*
Penicillin G	95.9 (90.1–98.5)	95.1 (87.2–98.4)	95.7 (91.0–98.1)	97.4 (93.9–99.0)	95.6 (91.5–98.0)	97.0 (93.8–98.7)	98.0 (94.4–99.4)	96.0 (94.4–97.6)	98.0 (96.1–99.9)	0.34 (-0.08–0.76)	1.202	0.115
Oxacillin	70.7 (61.2–78.8)	69.9 (58.3–79.6)	77.8 (70.3–84.0)	77.3 (70.6–82.9)	76.4 (69.6–82.2)	74.0 (67.6–79.7)	76.7 (69.3–82.9)	71.0 (65.5–76.5)	76.1 (68.9–83.3)	0.85 (-0.54–2.24)	0.300	0.382
Gentamicin	17.1 (10.7–25.9)	8.7 (3.7–17.5)	12.4 (7.7–19.1)	10.3 (6.4–15.9)	8.9 (5.2–14.4)	7.1 (4.2–11.6)	8.7 (5.0–14.5)	15.1 (10.2–20.0)	8.1 (3.2–13.0)	-1.16 (-2.24– -0.08)	-1.652	0.049
Rifampicin	8.9 (4.6–16.0)	7.6 (3.0–16.9)	9.7 (5.6–15.8)	9.0 (5.4–14.4)	3.6 (1.5–7.7)	7.6 (4.6–12.1)	3.3 (1.3–7.6)	8.9 (5.4–12.4)	5.4 (1.2–9.6)	-0.58 (-1.33–0.17)	-1.352	0.088
Ciprofloxacin	33.3 (24.7–43.1)	37.5 (26.8–49.5)	45.4 (37.4–53.7)	42.1 (34.9–49.6)	41.8 (34.6–49.4)	46.7 (39.9–53.7)	46.7 (38.8–54.8)	35.6 (29.3–41.9)	45.6 (37.5–53.7)	1.67 (0.23–3.11)	1.952	0.026
Levofloxacin	39.8 (30.7–49.6)	39.7 (28.9–51.6)	47.6 (39.5–55.8)	44.6 (37.3–52.1)	47.1 (39.7–54.7)	50.3% (43.4–57.2)	48.7 (40.8–56.7)	40.1 (34.2–46.0)	48.8 (41.3–56.3)	1.45 (0.05–2.85)	1.802	0.036
Moxifloxacin	22.0 (14.8–31.2)	25.0 (16.2–36.5)	30.8 (23.7–39.0)	20.2 (14.7–27.0)	26.7 (20.5–33.9)	30.5% (24.5–37.2)	29.3 (22.5–37.2)	23.0 (18.0–28.0)	28.8 (22.5–35.1)	0.96 (-0.25–2.17)	0.901	0.184
Cotrimoxazole	59.3 (49.6–68.4)	58.0 (46.2–69.0)	60.5 (52.3–68.2)	56.2 (48.7–63.5)	54.7 (47.2–62.0)	58.9 (52.0–65.5)	52.0 (44.0–59.9)	59.3 (53.8–64.8)	53.6 (46.5–60.7)	-0.95 (-2.31–0.41)	-1.502	0.067
Clindamycin	31.1 (22.8–40.7)	31.5 (21.6–43.4)	31.9 (24.7–40.0)	32.2 (25.6–39.5)	21.8 (16.1–28.8)	41.1 (34.5–48.0)	32.0 (25.0–39.9)	30.5 (24.8–36.2)	32.2 (24.1–40.3)	0.28 (-1.48–2.04)	1.201	0.115
Erythromycin	82.9 (74.5–89.0)	78.8 (67.9–86.8)	84.3 (77.4–89.4)	84.1 (78.0–88.8)	77.8 (71.0–83.5)	82.2 (76.4–86.9)	84.0 (77.3–89.0)	81.5 (77.5–85.5)	82.0 (76.9–87.1)	0.09 (-1.27–1.45)	0	0.500
Linezolid	0† (0–3.6)	0† (0–5.1)	0† (0–2.6)	0† (0–2.1)	0† (0–2.1)	0† (0–1.8)	0† (0–2.4)	0 (0–0)	0 (0–0)	0 (0–0)	0	0.500
Vancomycin	0† (0–3.6)	0† (0–5.1)	0%† (0–2.6)	0† (0–2.1)	0† (0–2.1)	0† (0–1.8)	0† (0–2.4)	0 (0–0)	0 (0–0)	0 (0–0)	0	0.500
Quinupristin/dalfopristin	0† (0–3.6)	0† (0–5.1)	0† (0–2.6)	0.4 (0.0–2.4)	0.4 (0.0–2.4)	1.0 (0.3–2.9)	0.7 (0.1–2.8)	0.0 (0.0–0.0)	0.9 (0.1–1.7)	0.15 (0.02–0.28)	2.103	0.018
Tetracycline	18.7 (12.1–27.5)	26.1 (17.0–37.7)	18.9 (13.1–26.4)	16.3 (11.4–22.6)	22.7 (16.9–29.6)	18.3 (13.5–24.3)	20.7 (14.8–28.2)	20.5 (16.0–25.0)	20.3 (14.4–26.2)	-0.04 (-1.05–0.97)	0	0.500
Tigecycline	0† (0–3.6)	0† (0–5.1)	0† (0–2.6)	0† (0–2.1)	0† (0–2.1)	0† (0–1.8)	0† (0–2.4)	0 (0–0)	0 (0–0)	0 (0–0)	0	0.500

Data are presented as % (95% CI). Linear regression was performed with year as continuous predictor and resistance percentage as outcome. Slope represents average annual change in percentage points. Predicted values and 95% CIs are derived from the regression model. Mann-Kendall Z and nominal *P*-values are shown for trend robustness. † For 0% or 100% events, the 95% CI upper or lower bound is estimated by the rule of three. ‡ After Bonferroni correction for 15 comparisons (α = 0.05/15 = 0.003), no trend reached statistical significance. The nominal *P*-values for gentamicin (0.049), ciprofloxacin (0.026), levofloxacin (0.036), and quinupristin/dalfopristin (0.018) are not significant after correction. Nitrofurantoin was excluded because it is not indicated for bloodstream infections

**Table 5:** Changes in resistance rates of *S. aureus* before, during, and after the COVID-19 pandemic

Antimicrobial agent	Pre-pandemic 2018–2019 (*N* = 137)	Pandemic 2020–2022 (*N* = 346)	Post-pandemic 2023–2024 (*N* = 92)	Z (trend)	*P* for trend
Penicillin G	86.9 (80.0–91.8)	92.7 (89.5–95.1)	100† (96.1–100)	4.12	< 0.001‡
Oxacillin	13.9% (8.8–20.9)	37.6 (32.6–42.8)	37.4 (27.8–47.9)	4.68	< 0.001‡
Gentamicin	7.3 (3.7–13.1)	3.8 (2.0–6.4)	3.3 (0.9–9.1)	-1.53	0.126
Rifampicin	2.2 (0.6–6.2)	1.7 (0.7–3.8)	0† (0–4.0)	-1.51	0.131
Ciprofloxacin	21.9 (15.5–29.9)	18.8 (14.9–23.4)	14.3 (8.3–23.3)	-1.55	0.121
Levofloxacin	21.9 (15.5–29.9)	18.8 (14.9–23.4)	13.1 (7.3–22.1)	-1.78	0.075
Moxifloxacin	21.2 (14.9–29.1)	17.9 (14.1–22.5)	7.6 (3.3–15.5)	-2.61	0.009
Cotrimoxazole	12.4% (7.6–19.4)	14.7 (11.3–19.0)	6.5 (2.7–13.8)	-1.23	0.219
Clindamycin	19.0 (13.0–26.7)	36.1 (31.2–41.3)	28.5 (19.9–38.9)	2.07	0.038
Erythromycin	46.0 (37.7–54.5)	63.6 (58.3–68.6)	55.0 (44.7–64.8)	2.16	0.031
Linezolid	0† (0–2.7)	0† (0–1.1)	0† (0–3.9)	–	–
Vancomycin	0† (0–2.7)	0† (0–1.1)	0† (0–3.9)	–	–
Quinupristin/dalfopristin	0† (0–2.7)	0† (0–1.1)	0† (0–3.9)	–	–
Tetracycline	24.1 (17.5–32.1)	21.7 (17.6–26.3)	19.7 (12.5–29.5)	-0.85	0.395
Tigecycline	0† (0–2.7)	0† (0–1.1)	0† (0–3.9)	–	–

Data are presented as % (95%CI). Cochran-Armitage test for linear trend across three ordered periods (pre-pandemic, pandemic, post-pandemic). † For 0% or 100% events, the 95% CI is estimated by the rule of three (3/n); for 100%, only the lower bound is estimated. ‡ After Bonferroni correction for 15 comparisons (α = 0.05/15 = 0.00333), only penicillin G and oxacillin remained statistically significant (*p* < 0.001). “–” indicates test not performed due to zero resistance in all periods. Nitrofurantoin was excluded because it is not indicated for bloodstream infections

**Table 6:** Changes in resistance rates of CoNS before, during, and after the COVID-19 pandemic

Antimicrobial agent	Pre-pandemic 2018–2019 (*N* = 172)	Pandemic 2020–2022 (*N* = 487)	Post-pandemic 2023–2024 (*N* = 355)	Z (trend)	*P* for trend
Penicillin G	95.9 (91.9–98.1)	96.1 (93.9–97.6)	97.5 (95.4–98.7)	1.15	0.25
Oxacillin	70.3 (63.2–76.7)	77.2 (73.3–80.8)	74.9 (70.2–79.2)	1.3	0.194
Gentamicin	13.4 (9.0–19.2)	10.5 (8.0–13.6)	7.6 (5.2–10.9)	-2.15	0.032
Rifampicin	8.1 (4.8–13.3)	7.4 (5.3–10.1)	5.6 (3.5–8.8)	-1.2	0.23
Ciprofloxacin	35.5 (28.6–43.0)	42.7 (38.4–47.1)	46.5 (41.4–51.7)	2.45	0.014
Levofloxacin	39.5 (32.4–47.1)	46.6 (42.3–51.0)	49.9 (44.7–55.0)	2.23	0.026
Moxifloxacin	23.3 (17.5–30.3)	25.7 (22.0–29.8)	30.1 (25.5–35.2)	1.85	0.064
Cotrimoxazole	58.7 (51.2–65.9)	57.1 (52.7–61.4)	56.1 (50.9–61.2)	-0.58	0.562
Clindamycin	30.8 (24.3–38.2)	28.3 (24.5–32.5)	37.2 (32.3–42.4)	1.78	0.075
Erythromycin	81.4 (74.9–86.6)	81.9 (78.3–85.1)	83.1 (78.9–86.7)	0.54	0.589
Linezolid	0† (0–2.1)	0† (0–0.8)	0† (0–1.0)	–	–
Vancomycin	0† (0–2.1)	0† (0–0.8)	0† (0–1.0)	–	–
Quinupristin/dalfopristin	0† (0–2.1)	0.4 (0.1–1.5)	0.8 (0.3–2.5)	1.58	0.114
Tetracycline	22.1 (16.5–28.9)	19.3 (16.0–23.0)	19.4 (15.5–24.0)	-0.81	0.418
Tigecycline	0† (0–2.1)	0† (0–0.8)	0† (0–1.0)	–	–

Data are presented as % (95% CI). Cochran-Armitage test for linear trend across three ordered periods.† For 0% events, the upper bound of the 95% CI is estimated by the rule of three (3/n). ‡ After Bonferroni correction for 15 comparisons (α = 0.05/15 = 0.003), no comparison remained statistically significant. The nominal *P*-values for gentamicin (0.032), ciprofloxacin (0.014), and levofloxacin (0.026) are not significant after correction. “–” indicates test not performed due to zero resistance in all periods. Nitrofurantoin excluded. Nitrofurantoin was excluded because it is not indicated for bloodstream infections

## Results

### Patient baseline characteristics

As shown in Table 1, this study included 575 patients with *S. aureus* and 1,014 with CoNS. No significant sex difference was observed (*χ²* = 1.430, *P* = 0.232). Age distribution differed significantly (*χ²* = 120.679, *P* < 0.001). *S. aureus* infections predominated in young and middle-aged adults (18–65 years, 51.8%), whereas CoNS infections were more frequent in minors (0–17 years, 31.3%) and the very elderly (≥ 80 years, 13.7%).

### Strains changes by year

**Fig. 2:**
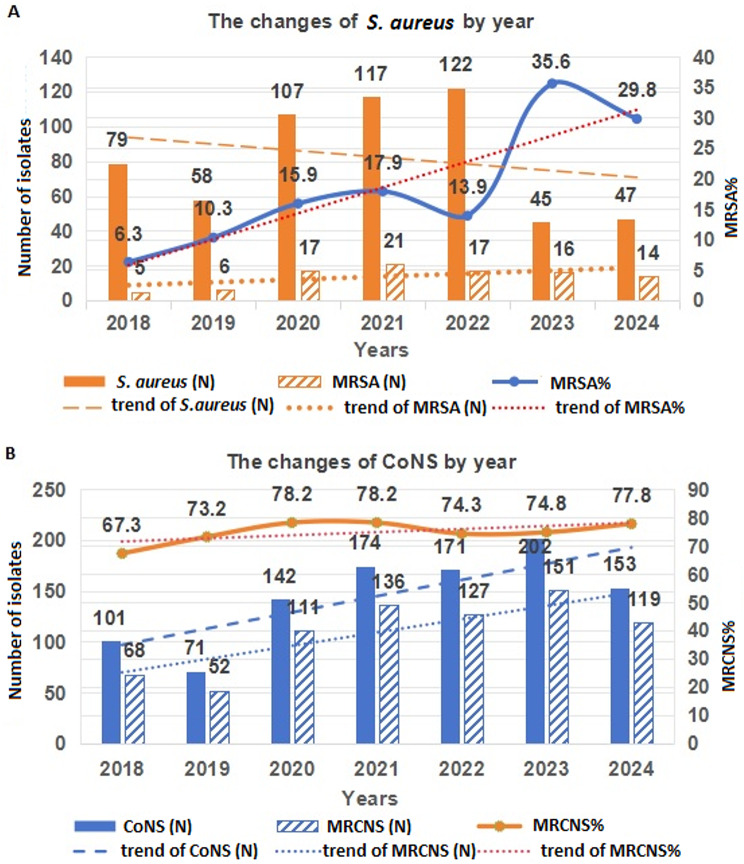
Annual number of staphylococcal isolates and methicillin resistance rates (2018–2024). **(A)** *S. aureus* and MRSA. Bars show the annual number of total *S. aureus* (solid bars) and MRSA (hatched bars) isolates. The line with markers shows the MRSA percentage (right y-axis). The dashed line indicates the linear trend. **(B)** CoNS and MRCNS. Bars show the annual number of total CoNS (solid bars) and MRCNS (hatched bars) isolates. The line with markers shows the MRCNS percentage (right y-axis). The dashed line indicates the linear trend

As shown in Fig. 2, the number of *S. aureus* isolates peaked during 2020–2022 and declined thereafter. MRSA proportion remained high from 2023 onward. CoNS isolates showed a linear upward trend from 2020 to 2023, with a slight decrease in 2024. MRCNS proportion consistently exceeded 70% throughout.

### Comparison of overall drug resistance between *S. aureus* and CoNS

Table 2 presents resistance rates. CoNS exhibited significantly higher resistance than *S. aureus* to oxacillin (75.2% vs. 30.5%, *P* < 0.001), ciprofloxacin (42.8% vs. 17.5%, *P* < 0.001), levofloxacin (46.5% vs. 17.4%, *P* < 0.001), cotrimoxazole (57.0% vs. 10.8%, *P* < 0.001), and erythromycin (82.2% vs. 56.1%, *P* < 0.001). After Bonferroni correction (α = 0.003), these differences remained significant. No resistance to linezolid, vancomycin, or tigecycline was detected. Quinupristin/dalfopristin showed no resistance in *S. aureus* and minimal resistance in CoNS (0.5%).

### Annual drug resistance trends

After linear regression analysis, no antimicrobial agent in either *S. aureus* or CoNS showed a statistically significant trend in resistance over time after Bonferroni correction (α = 0.003). Nominal trends were observed for penicillin G in S. aureus (slope 2.15% per year, *P* = 0.049) and for gentamicin, ciprofloxacin, levofloxacin, and quinupristin/dalfopristin in CoNS (nominal *P* < 0.05), but none survived correction for multiple comparisons. Detailed slopes, predicted values, and confidence intervals are presented in Tables 3 and 4.

### Drug resistance changes before, during, and after the COVID-19 pandemic

For *S. aureus* (Table 5), the Cochran-Armitage test for linear trend across the three periods (pre-pandemic, pandemic, post-pandemic) revealed statistically significant increasing trends for penicillin G (Z = 4.12, *P* < 0.001) and oxacillin (Z = 4.68, *P* < 0.001). After Bonferroni correction for 15 comparisons (α = 0.003), these two agents remained significant. Clindamycin (Z = 2.07, *P* = 0.038) and erythromycin (Z = 2.16, *P* = 0.031) showed nominal increasing trends, but these did not survive correction. No other antimicrobial agent exhibited a significant trend. Resistance to linezolid, vancomycin, and tigecycline remained zero throughout all periods.

For CoNS (Table 6), the Cochran-Armitage test did not identify any statistically significant linear trend after Bonferroni correction. Nominal *P*-values below 0.05 were observed for gentamicin (Z = -2.15, *P* = 0.032), ciprofloxacin (Z = 2.45, *P* = 0.014), and levofloxacin (Z = 2.23, *P* = 0.026), but none reached the corrected significance threshold (α = 0.003). Resistance rates for penicillin G and oxacillin remained consistently high across periods without significant trends.

## Discussion

This study systematically analyzed the clinical distribution and antimicrobial resistance trends of *S. aureus* and CoNS in blood isolates from 2018 to 2024. Our findings reveal differences between the two groups in population distribution and resistance profiles, as well as temporal associations coinciding with the COVID-19 pandemic. However, due to the retrospective single-center design and the lack of adjustment for potential confounders, these associations should not be interpreted as causal.

### Population distribution

This study indicates that *S. aureus* infections were more frequently observed among young and middle-aged patients (18–65 years), whereas CoNS infections were proportionally higher in pediatric patients (0–17 years) and individuals aged ≥ 80 years. Previous research confirms that CoNS are predominant pathogens in extreme age groups such as newborns, particularly among preterm and low-birth-weight infants [6]. Recent multicenter retrospective studies focusing on *S. aureus* bacteremia in the oldest old (≥ 85 years) and in frail nursing home residents (mean age > 80 years) have provided supportive evidence regarding its unique epidemiology, high mortality, and the critical impact of functional status beyond chronological age alone [7, 8]. These differences are likely associated with host immune status, underlying comorbidities, and healthcare exposures—such as invasive procedures that are more common in elderly and pediatric populations [9]. We did not have data to test these hypotheses directly. Age therefore stands out as one of the factors distinguishing the epidemiological profiles of these two staphylococcal infections.

### Comparison of resistance between *S. aureus* and CoNS

CoNS isolated from blood cultures exhibited significantly higher overall resistance rates to most tested antimicrobial agents than *S. aureus*, particularly to fluoroquinolones, macrolides (erythromycin), and folate pathway inhibitors (cotrimoxazole). This may be related to their role as skin commensals with greater opportunity to acquire resistance genes [9–11]. However, we cannot rule out the influence of contamination misclassification on these comparisons. Fortunately, linezolid, vancomycin, and tigecycline remained 100% susceptible in vitro against both bacterial groups. We refrain from calling them “reliable last-line options” because clinical efficacy requires correlation with outcomes, which we did not assess.

### Annual resistance trends

For *S. aureus*, resistance to penicillin G increased by an average of 2.15% per year (95% CI: 0.62–3.68), and oxacillin by 3.92% per year (95% CI: 1.15–6.69), which is consistent with the highly prevalent production of penicillinase in this species [12, 13]. For CoNS, nominal increases in ciprofloxacin (1.67%/year, 95% CI: 0.23–3.11) and levofloxacin (1.45%/year, 95% CI: 0.05–2.85) were observed, possibly driven by selective pressure from the extensive clinical use of these agents [14, 15]. A nominal decrease in gentamicin in CoNS was noted (–1.16%/year, 95% CI: − 2.24 to − 0.08), which may reflect changes in aminoglycoside prescribing practices or variations in resistance gene stability [16–18]. However, after Bonferroni correction for 15 comparisons (α = 0.003), no annual trend remained statistically significant for either bacterial group. These findings suggest that while certain resistance phenotypes may have drifted upward over the seven-year period, the changes were modest and not robust to correction for multiple testing. The Mann-Kendall test yielded consistent nominal *P*-values (data shown in Tables 3 and 4).

### Temporal associations with the COVID-19 pandemic

The COVID-19 pandemic undoubtedly served as a major disruptive event, significantly altering the epidemiology of bacterial resistance [19–22]. Our data demonstrate that during and after the pandemic period, *S. aureus* exhibited higher resistance rates to penicillin G, oxacillin, clindamycin, and erythromycin compared with pre-pandemic levels. After Bonferroni correction (α = 0.003), only the increasing trends for penicillin G and oxacillin remained statistically significant (both *P* < 0.001). The nominal increases for clindamycin (*P* = 0.038) and erythromycin (*P* = 0.031) did not survive correction. For CoNS, higher resistance rates to ciprofloxacin and clindamycin were observed but none reached statistical significance after correction (ciprofloxacin *P* = 0.014, clindamycin *P* = 0.075, corrected α = 0.003). Possible contributing factors include pandemic-related changes in antibiotic consumption, infection control practices, and patient case mix [19–22]. Because we did not measure these variables, causality cannot be established.

### Epidemiological implications of strain distribution changes

The isolation rates of *S. aureus* and MRSA from blood cultures peaked during 2020–2022 and declined thereafter. However, the proportion of MRSA among all *S. aureus* isolates remained persistently high from 2023 onward, indicating that the relative prevalence of MRSA has not diminished despite the decrease in absolute numbers. Concurrently, CoNS exhibited a linear upward trend in total isolate numbers, with MRCNS proportions consistently exceeding 70%. These observations indicate that CoNS constitute a substantial and highly resistant reservoir of pathogens in this setting.

### Impact of laboratory methodology change

The introduction of MALDI-TOF in 2019 could theoretically affect species identification. We compared resistance rates in 2018 and 2019 and found no abrupt discontinuity (data not shown), but subtle effects cannot be excluded.

### Clinical implications

The in vitro activity of linezolid, vancomycin, and tigecycline is informative but does not replace clinical outcome data. These agents should be used judiciously based on susceptibility testing.

## Limitations

This study has several important limitations that should be considered when interpreting the findings. (a) Retrospective, single-center design limits generalizability. (b) CoNS contamination vs. infection: misclassification possible despite clinical criteria. (c) Temporal association vs. causality: no adjustment for confounders (antibiotic use, ICU occupancy, infection control). (d) Laboratory methodology change: MALDI-TOF introduced in 2019; no discontinuity detected, but bias possible. (e) CoNS species aggregation: species-level data unavailable. (f) Missing clinical data: no information on prior antibiotics, comorbidities, outcomes, or antimicrobial consumption. (g) Multiple comparisons: Bonferroni correction is conservative; some true associations may be missed (type II error). (h) In vitro data only: does not guarantee clinical efficacy. (i) Sample size variations: annual fluctuations may affect stability of estimates. Despite these limitations, this seven-year analysis provides valuable insight into staphylococcal resistance trends in a large regional medical center.

## Conclusion

The antimicrobial resistance profile of staphylococci isolated from blood cultures has shown concerning trends, with temporal increases in resistance to several agents observed during the COVID-19 pandemic period. After Bonferroni correction, only S. aureus penicillin G and oxacillin showed significant pandemic-period increases, while CoNS trends were not significant. Given persistently high methicillin resistance, empirical oxacillin use should be reconsidered. Ongoing surveillance and prospective studies with clinical outcomes are needed.

## Data Availability

The raw data supporting the findings of this study are available from Mianyang Central Hospital. But restrictions apply to the availability of these data, which were used under licence for the current study and so are not publicly available. The raw data are, however, available upon request and with the permission of Mianyang Central Hospital.

## Abbreviations

*S. aureus*: *Staphylococcus aureus*
CoNS: Coagulase-negative staphylococci
CIs: Confidence intervals
χ^2^: Chi-square test
BSIs: Bloodstream infections
MRCNS: Methicillin-Resistant CoNS
MRSA: Methicillin-Resistant S. aureus
NS: Not significant
CLSI: Clinical and Laboratory Standards Institute
MALDI-TOF: Matrix-assisted laser desorption/ionization time-of-flight
